# Functional Coupling of a Nematode Chemoreceptor to the Yeast Pheromone Response Pathway

**DOI:** 10.1371/journal.pone.0111429

**Published:** 2014-11-21

**Authors:** Muhammad Tehseen, Mira Dumancic, Lyndall Briggs, Jian Wang, Amalia Berna, Alisha Anderson, Stephen Trowell

**Affiliations:** CSIRO Food Futures National Research Flagship & CSIRO Ecosystem Sciences, Australia, PO Box 1700, Canberra, ACT 2601, Australia; Rutgers University, United States of America

## Abstract

Sequencing of the *Caenorhabditis elegans* genome revealed sequences encoding more than 1,000 G-protein coupled receptors, hundreds of which may respond to volatile organic ligands. To understand how the worm's simple olfactory system can sense its chemical environment there is a need to characterise a representative selection of these receptors but only very few receptors have been linked to a specific volatile ligand. We therefore set out to design a yeast expression system for assigning ligands to nematode chemoreceptors. We showed that while a model receptor ODR-10 binds to *C. elegans* Gα subunits ODR-3 and GPA-3 it cannot bind to yeast Gα. However, chimaeras between the nematode and yeast Gα subunits bound to both ODR-10 and the yeast Gβγ subunits. *FIG2* was shown to be a superior MAP-dependent promoter for reporter expression. We replaced the endogenous Gα subunit (*GPA1*) of the *Saccharomyces cerevisiae (ste2Δ sst2Δ far1Δ)* triple mutant (“Cyb”) with a Gpa1/ODR-3 chimaera and introduced ODR-10 as a model nematode GPCR. This strain showed concentration-dependent activation of the yeast MAP kinase pathway in the presence of diacetyl, the first time that the native form of a nematode chemoreceptor has been functionally expressed in yeast. This is an important step towards *en masse* de-orphaning of *C. elegans* chemoreceptors.

## Introduction

G-protein coupled receptors (GPCRs) comprise the largest and most diverse superfamily of eukaryotic proteins. They are integral membrane proteins, involved in a variety of functions including vision, smell, taste and nociception as well as cellular recognition and communication processes. Structurally, GPCRs are characterised by having an amino-terminal extracellular domain, a carboxyl-terminal intracellular domain and seven hydrophobic transmembrane domains: functionally, GPCRs signal through heterotrimeric G-proteins [1], [2]. GPCRs may be activated by a wide variety of ligands, including peptide and non-peptide neurotransmitters, hormones, growth factors, odorant molecules and also by light [3].

The anatomy of the olfactory system of *Caenorhabditis elegans* is much simpler than that of mammals, comprising only thirteen pairs of sensory neurons and a limited neural capacity to process olfactory information. Nevertheless, behavioural experiments have shown that *C. elegans* shows very high sensitivity to more than 100 volatile compounds from many chemical classes [4], [5].

The *C. elegans* genome encodes over 1,200 putative chemosensory receptors [6]-[8], which belong to the same GPCR superfamily as mammalian odorant receptors [5]. Until very recently only three of these receptors have been linked to specific ligands. ODR-10, a member of the *str* family of GPCRs, which is expressed predominantly in AWA neurons [9], [10] binds to and mediates chemotaxis towards the volatile ligand 2, 3-butanedione (diacetyl) [10]. The SRBC receptors SRBC-64 and SRBC-66 were shown, jointly, to respond to non-volatile ascaroside components of the dauer pheromone [11]. Both of these discoveries were the result of painstaking genetic screens; in the case of ODR-10 a screen for chemosensory mutants and in the case of SRBC-64/-66 a screen for defects in the dauer pathway. In the latter case, the search was facilitated both by prior identification of the dauer pheromone [12] and the knowledge that it modulates the biology and developmental fate of the organism in a fundamental and uniquely obvious manner. Knowledge of the ligand-specificity, affinity and chemoreceptive tuning of a larger subset of nematode chemoreceptors, particularly those involved in olfaction, would be helpful in understanding nematode chemosensation. [13] have recently carried out an elegant RNAi screen and identified 194 odorant receptor genes linked to 11 odorants. However, further analysis of many of these candidate genes to confirm ligand-receptor pairs through phenotypic means is not practical. High throughput screening assays based on heterologous expression of odorant receptors represent an alternative strategy.

Zhang *et al* [10] expressed *C. elegans* ODR-10 in a mammalian cell line and observed a transient elevation in intracellular Ca^2+^ levels in response to µM concentrations of diacetyl and some other ligands. However, the percentage of cells responding to the ligand in that study was low, requiring statistically based methods such as flow cytometry for an adequate readout. On the other hand, yeast's rapid growth, its ease of manipulation and the availability of different reporter markers might make it a better host for de-orphaning *C. elegans* chemosensory receptors.

Yeast-based heterologous assays have been widely used to functionally express and de-orphan a wide range of non-olfactory GPCRs [14]–[16] and have also been applied for de-orphaning mammalian olfactory receptors [16], [17]. Minic *et al* [18] used a luciferase reporter to demonstrate the activation of human and rat olfactory receptors heterologously expressed in the yeast, *Saccharomyces cerevisiae* but, in our hands, this unmodified system could not be used to express *C. elegans* olfactory receptors (ORs) (unpublished). Typically, yeast-based systems must be customised in order to successfully express receptors from other organisms. Necessary modifications have variously included making chimaeric receptors, replacing or modifying Gα subunits and deleting some of the yeast proteins that modulate the GPCR signalling pathway. In this study, we took the same general approach as for mammalian pharmacological receptors, specifically customising brewer's yeast, *Saccharomyces cerevisiae,* so as to express the *C. elegans* olfactory GPCR ODR-10.

To develop a heterologous expression assay, we need Gα subunits that can couple the GPCR functional classes of interest to the host yeast MAP-kinase pathway via yeast Gβγ. The *C. elegans* genome encodes 21 different Gα subunits, of which, six are expressed in olfactory neurons, along with two Gβ and two Gγ genes [19], [20]. Of the six olfactory Gα subunits, ODR-3, is an important stimulatory mediator and is sufficient for odorant detection [21]. GPA-3 also supports stimulatory signalling in AWA and AWC olfactory neurons, acting redundantly with ODR-3 [5], [21], [22].

Based on genetic studies, *C. elegans* ODR-10's ligand and its interactions with Gα subunits ODR-3 and GPA-3 have been determined [9], [10], [22]. We therefore used ODR-10 as a model receptor to optimise coupling of nematode ORs to the yeast MAP kinase signalling pathway. We showed that, unlike a mammalian olfactory Gα, unmodified ODR-3 and GPA-3 can't activate the signalling pathway but their chimaeras with the yeast Gα subunit, GPA-1, are competent to do so. We also used a split-ubiquitin yeast two-hybrid screen to demonstrate physical interaction between ODR-10 and the yeast/worm Gα chimaeras. Additionally, we compared the signal to noise properties of two pheromone responsive promoters, *FUS1* and *FIG2,* in driving expression of reporter genes. Based on these elements, we developed and optimised a functional assay for diacetyl-sensing, demonstrating that it is robust in a 96 well assay plate format, the first time this has been achieved with a *C. elegans* chemoreceptor.

## Materials and Methods

### Yeast strains and media

The *S. cerevisiae* host strain, BY4741 *ste2Δ* single mutant (Euro1) (BY4741; MATa; *his3Δ1; leu2Δ0; met15Δ0; ura3Δ0; YFL026w::kanMX4*) was used to generate the ‘Cyb’ yeast mutant. We sequentially knocked out *SST2* and *FAR1* genes using homologous recombination by inserting different antibiotic markers at the gene loci. For *SST2* knockout, the nourseothricin N-acetyl-transferase disruption cassette was amplified by PCR using the *SST2* targeting primers: SST2F KO and SST2R KO (Table S1). These primers were derived from pAG25[23]. The resulting PCR product was used to transform the Euro1 strain which was selected on YPD plates containing 100 µg/ml nourseothricin (clonNAT). Correct integration of the cassette into the *SST2* locus was verified by colony PCR using the primers SST2F Diag RIF2 and SST2R Diag pAG25 (Table S1) flanking the *SST2* coding sequence. Following PCR, sequencing was performed to confirm the integration. For *FAR1* knockout, the hygromycin B phopsphotransferase disruption cassette was amplified by PCR using the *FAR1* targeting primers: FAR1F KO and FAR1R KO primers (Table S1). These primers were derived from pAG32 [23]. The resulting PCR product was used to transform the Euro1 *sst2Δ* mutant strain, which was selected on YPD plates containing 300 µg/ml hygromycin B. Correct integration of the cassette into the *FAR1* locus was verified by colony PCR using the primers FAR1F Diag SSY5 and Far1F Diag SSY5 (Table S1) flanking the *FAR1* coding sequence and sequencing. Sequential disruption of the *SST2* and *FAR1* genes resulted in generation of the Cyb (*ste2::kanMX4 sst2::natMX4 far1::hphMX4*) yeast triple mutant.

Yeast strain YGS5 (YPH499 *gpa1::hisGste11^ts^*): YPH499 (*MAT* **a** *ura3-52 lys2-801^am^ ade2-101^oc^ trp1-Δ63his3-Δ200leu2-Δ1*) also used in this study was kindly provided by Assoc Prof Yuqi Wang, Saint Louis University, USA. The NMY51 {*Mata his3Δ200 trp1-901, 112 ade2 LYS5:(LexAop)_4_-HIS3 ura3::(LexAop)_8_-LacZ ade2::::(LexAop)_8_-ADE2 GAL4}* reporter strain used for split ubiquitin membrane yeast-two hybrid assay was purchased from Dualsystems Biotech Switzerland.

### Construction of plasmids for yeast chromosome substitution

The plasmids used for substitution at the yeast *GPA1* chromosomal locus were *GPA1/odr-3* chimaera and *GPA1/gpa-3* chimaera. In *GPA1/odr-3* and *GPA1/gpa-3* chimaeras the 5 carboxyl-terminal amino acids residues of the endogenous yeast Gα subunit (Gpa1) were substituted by that of *C. elegans* ODR-3 and GPA-3 Gα subunits. Two DNA fragments encoding the *GPA1/odr-3* and *GPA1/gpa-3* chimaeras and downstream region (*GPA1* terminator) of *GPA1* open reading frame (ORF) were amplified from Euro1 with the following primer pairs: Gpa1F *Kpn*I and Gor3Chi *Apa*I (*GPA1/odr-3* chimaera), Gpa1F *Kpn*I and Gga3Chi *Apa*I (*GPA1/gpa-3* chimaera), Gpa1tF *Apa*I and Gpa1tR *Eco*RI (Table S1). The *Kpn*I*-Apa*I *GPA1/odr-3* and *GPA1/gpa-3* gene fragments were inserted separately into pUC57 along with the *Apa*I*-Eco*RI *GPA1t* fragment, resulting in the plasmids pUC57- *GPA1/odr-3* chimaera-*GPA1t* and pUC57-*GPA1/gpa-3* chimaera-*GPA1t*. A 1.5 kb fragment containing the *URA3* gene with its promoter and terminator was amplified from *Pichia pastoris* using URA3F *Eco*R1 and URA3R *Bam*HI primers (Table S1) and cloned into pUC57- *GPA-1/odr-3* chimaera-*GPA-1t* and pUC57-*GPA1/gpa-3* chimaera-*GPA1t* resulting in the pUC57-*GPA1/odr-3* chimaera-*GPA1t*-*URA3* and pUC57-*GPA1/gpa-3* chimaera-*GPA1t*-*URA3* plasmids. Finally an approximately 600 bp fragment adjacent to the *GPA1* gene was amplified from Euro1 for use as flanking fragment using the Nem1F *Bam*HI and Nem1R *Spe*I primers (Table S1) and cloned into both plasmids downstream of the *URA3* marker. The resulting plasmids were designated *GPA1/odr-3* chimaera-PpURA3 and *GPA1/gpa-3* chimaera-PpURA3 (Figure S1).

### Construction of expression plasmids

The full-length coding sequence of the *C. elegans* olfactory receptor, ODR-10 was expressed under the control the *PGK1* promoter. We amplified a 720 bp fragment of the *PGK1* promoter from Euro1, using primers PGKprom-719FWD-EcoRI and PGKprom-1REV-ApaI Table S1), digested with *Eco*RI and *Apa*I and cloned into pESC-HIS (Stratagene) that had been digested with *Apa*I and *Eco*RI to remove both *GAL1* and *GAL10* promoters. This vector is designated as pPGK-HIS. The ccdB primers (Table S1) were used to amplify a 1.6 kb fragment from pYES-DEST52 (Invitrogen) that contains the AttR1/ccdB/AttR2 cassette, which was digested with *Apa*I and *Xho*I and cloned into pPGK-HIS immediately downstream of the *PGK1* promoter. This vector is designated pDEST PGK-HIS (Figure S2). *Odr-10* was recombined into pDEST PGK-HIS using a standard LR Clonase (Invitrogen) reaction and its presence was confirmed by restriction digest. The non-functional control version of *odr-10* was constructed using site directed mutagenesis to introduce the histidine to tyrosine substitution at residue 110 (H110Y) [24] and this was confirmed by sequencing. *Odr-10* H110Y was then recombined into pDEST PGK-HIS using a standard LR Clonase (Invitrogen) reaction and its presence was confirmed by restriction digest. For cellular localisation studies, *odr-10* containing GFP^2^ in the third intracellular loop was cloned into pDEST PGK-HIS by LR Clonase mediated recombination reaction and its presence was confirmed by sequencing. *FIG2:lacZ* reporter construct was made by amplifying 587 bp fragment of the *FIG2* promoter from Euro1, using primers Fig2F BamHI and Fig2R NcoI and 3072 bp fragment of *lacZ* gene using primers LacZF NcoI and LacZR SpeI (Table S1), digested with *Nco*I and ligated both PCR fragments. 3659 bp *FIG2:lacZ* fragment amplified from ligation using primers Fig2F BamHI and LacZR SpeI, digested with BamHI and SpeI and cloned into pESC-LEU (Stratagene).

For expression of nematode Gα proteins in the yeast system, *odr-3* and *gpa-3* and their chimaeras were cloned downstream of the *GPA1* promoter into a pESC-URA (Stratagene) plasmid. To construct this plasmid, we amplified a 702 bp fragment of *GPA1* from Euro1, using primers Gpa1pF and Gpa1pR (Table S1), digested it with *Age*I and *Not*I and cloned it into pESC-URA digested with *Age*I and *Not*I to remove the *GAL10* promoter. The vector is designated as pGPA1-URA. Full length ORFs of *GPA1*, *odr-3*, *gpa-3*, *GPA1/odr-3* Chimaera and *GPA-1/gpa-3* Chimaera were amplified using the primers shown in Table S1 and cloned into the pGPA1-URA plasmid immediately downstream of the *GPA1* promoter at *Not*I and *Cla*I restriction sites which was verified by sequencing.

### Yeast transformations and media

Yeast strains were transformed using the lithium acetate method [25], grown in YPD medium [containing 1% yeast extract, 2% peptone and 2% glucose] or synthetic drop out medium (SD medium) [containing 0.67% yeast nitrogen base without amino acids and 2% glucose]. The SD medium was supplemented with the appropriate amino acids depending on the targeted selectable marker. For solid media, 2% agar was added.

### Protein-Protein interaction assays using Split-ubiquitin yeast two-hybrid system

The split-ubiquitin yeast two-hybrid system [26] was used to investigate interactions among ODR-10, ODR-3, GPA-3, Gpa1/ODR-3 chimaera and Gpa1/GPA-3 chimaera. Vectors and yeast strain were supplied in the DUALmembrane pairwise interaction kit (Dualsystems Biotech, Zürich, Switzerland). The full length *odr-10* cDNA was cloned into the pBT3-STE plasmid encoding the C-terminal half of ubiquitin (Cub) such that it fused to the C-terminus of the *odr-10* (ODR10-Cub). The full-length *odr-3*, *gpa-3*, *GPA1/odr-3* chimaera and *GPA-1/gpa-3* chimaera cDNAs were cloned into pPR3-STE which encodes the mutated N-terminal half of ubiquitin (NubG) such that it was fused to the C-termini of the G-proteins (Gene-NubG) and pPR3-N plasmids such that the NubG was fused to the N-termini of the G-proteins (NubG-Gene). All cDNAs were cloned into *Sfi*I restriction sites. cDNA sequences were confirmed by DNA sequencing. The interactions were tested by co-transforming plasmids into reporter yeast strain (NMY51). Interaction was determined by assessing the growth of transformants on medium lacking histidine and was confirmed using a β-galactosidase assay (HTX Kit: Dualsystems Biotech, Zürich, Switzerland).

### Confocal microscopy

Yeast transformants expressing ODR-10-GFP^2^ were grown in SD media without histidine at 30°C overnight and the cells were inoculated into 10 ml of the same media to give an initial Abs_600_ = 0.025. The cells were again grown at 30°C overnight on a rotary shaker at 180 rpm. For microscopy, a 20 µl drop of yeast culture was placed on a clean glass slide and stained with 0.01% Evans Blue (Sigma) in culture medium, for 1 min, covered with a cover slip and observed with a Leica SP2 confocal microscope (Germany) with 488 nm excitation and imaging 510 nm for GFP^2^emission and 660 nm for Evans Blue emission.

### Ligand Assay

Yeast transformants (Cyb Gpa1/ODR-3 chimaera or Cyb Gpa1/GPA-3 chimaera) containing the *PGK1*:ODR-10 receptor and *FIG2:lacZ* reporter were grown at 30°C overnight on a rotary shaker at 180 rpm in 10 ml of synthetic liquid dropout medium, containing glucose (2%) but lacking uracil, histidine and leucine. For ligand assays the cells were inoculated into the same media to give an Abs_600_ = 1. 1 ml culture was used for the ligand assay in eppendorf tubes. All ligand solutions were diluted in water. Cells were incubated with ligand for seven hrs by placing tubes horizontally at 30°C on a rotary shaker at 110 rpm. The β-galactosidase assay was performed using the chromogenic substrate ortho-nitrophenyl-β-D-galactopyranoside (ONPG) and absorbance was measured at 414 nm.

### GC-MS measurements

In order to assess the rate of diacetyl degradation in this system, 8 ml of cultures were prepared as described above and placed into 10 ml vials, incubation times were 0, 1, 2, 4, 5, 6 or 7 hours 35°C with shaking (200 rpm). Headspace extraction was carried out for 2 minutes with a solid phase micro-extraction SPME fibre (Aldrich, Bellefonte, PA) composed of fused silica partially cross-linked with 65 µm polydimethylsiloxane/divinylbenzene (PDS/DVB). After absorption, headspace volatiles were transferred to the injection port of gas chromatograph (GC), which was equipped with a 0.8 mm i.d. splitless glass liner, at 250°C. Desorbed volatile compounds were separated in a Varian 3800 GC, equipped with a 30 m×0.25 mm, 0.25 µm film thickness ZB-5MS fused silica capillary column. The oven temperature was programmed to rise from 50°C to 180°C at 5°C min^−1^, followed by a ramp of 30°C min^−1^ up to 240°C. The GC column output was fed into a Varian 1200 mass selective detector (mass spectrometer). The GC-MS transfer line was heated at 250°C with the flow rate of the He carrier gas set to 1 ml min^-1^. Mass spectrometry was performed in electron impact mode at 70 eV over the scan range 35–350 *m/z.* Three repetitions of each sample was analysed and areas under the curve were calculated for relevant peaks.

### Assay for large scale screening

For the high-throughput assay, fresh yeast cells (Cyb Gpa1/ODR-3 or Cyb Gpa1/GPA-3 chimaera/*PGK1*:ODR-10 containing *FIG2:lacZ* reporter) were grown in SD medium containing glucose (2%) but lacking uracil, histidine and leucine in 96-well deep well plate at 30°C with shaking at 900 rpm for 24 hrs. Cells were diluted as 1/10 dilution in fresh 1 ml medium into new 96-well deep plate (100 ul cells+900 ul medium) and induced by adding 50 µl of 10 mM odorant in a fume hood to give a final of ligand concentration of 500 µM and incubated at 30°C with shaking at 900 rpm for 17–20 hrs. Cells were pelleted at 1500×g for 5 min and the supernatant was discarded by shaking out in a fume hood and draining briefly onto absorbent paper. The cell pellet was resuspended in 120 µl lysis buffer (100 mM sodium phosphate buffer (82 mM Na_2_HPO_4_ and 12 mM NaH_2_PO_4_), pH 7.5, containing 0.1% sodium dodecyl sulfate) [27] and incubated at 30°C for 10 min at 900 rpm. At the end 40 µl of 2.5 mM ONPG (10 mg ONPG dissolved in 130 µL dimethyl formamide is 250 mM, then dilute 1/100 in water for final 2.5 mM) was added and incubated at room temperature for 10 min [27] to develop a color. Reaction was stopped by adding 80 µl of 1 M Na_2_CO_3_. Cells were pelleted and 100 ul supernatant was carefully transferred to a clear 96-well normal plate and absorbance was read at 414 nm.

## Results and Discussion

### Construction of the Cyb yeast strain

Selective and sensitive bioassay of functionally expressed GPCRs is facilitated by yeast strains that respond to receptor activation by exhibiting a visible response, rather than cell cycle arrest. However, to date, this has not been achieved for *C. elegans* chemosensory GPCRs. Based on approaches that have been successful with mammalian GPCRs, several modifications were made to optimise the system and increase the coupling of heterologous GPCRs to the pheromone pathway. We used a haploid yeast strain *ste2Δ* single mutant derived from BY4741 (obtained from EUROSCARF, Germany). The *STE2* mutation is important because the presence of an endogenous pheromone receptor can diminish the ability to detect signalling from foreign GPCRs, apparently by sequestering G proteins into refractory pre-activation complexes [28]. The *FAR1* gene was deleted to avoid cell death due to overstimulation of this transduction pathway [14], [29]–[31]. The ability to detect signalling was further enhanced by deleting the regulator of G-protein (RGS), *SST2*, which results in increased gain in the MAP kinase transduction cascade [14],[32]. The resulting *ste2Δ far1Δ sst2Δ* triple mutant was designated as ‘Cyb’.

It is assumed that heterologous GPCRs, such as ODR-10, introduced into *S. cerevisiae* do not interfere with or substitute for any of the three known yeast GPCRs, namely the STE2 and STE3 receptors for the alpha- and a-mating factors, respectively and the glucose receptor GPR1 [33]. ODR-10 shows no homology to these endogenous GPCRS. A BLAST search, using the ODR-10 sequence to query PubMed and *S. cerevisiae* genome databases, returned no plausible ODR-10 homologues in yeast.

### Characterisation of yeast/*C. elegans* Gα chimaeras

In order for heterologous GPCRs to function in the yeast MAP kinase transduction cascade, they must efficiently activate G-proteins. Therefore, we tested whether, like some plant, mammalian and rat Gα-proteins [34]–[36], *C. elegans* ODR-3 and GPA-3 are capable of binding to the yeast β/γ complex, by assessing their ability to complement the growth arrest phenotype of YGS5 a *gpa1Δ* mutant strain [37], [38]. A yeast *gpa1* deletion in a wild-type background is effectively lethal, because absence of GPA1 leaves yeast Gβγ subunits free to bind to downstream MAP kinase signalling elements, leading to growth arrest [37], [38]. However a mutation in *ste11*, a gene downstream of MAP3K, can compensate for the *gpa1* deletion by blocking the yeast MAP kinase cascade. The YGS5 yeast strain carries a temperature sensitive mutation in *ste11* in addition to the *gpa1* knockout. In this background, the *gpa1* deletion is viable at higher temperatures (34°C) because activation of the MAP-kinase cascade by free Gβγ is blocked by thermal inactivation of the STE11 protein. The strain can therefore be maintained at the higher temperature. However, when YGS5 is switched to the lower temperature (24°C), STE11 folds properly, MAP-kinase signalling is restored and cell cycle arrest occurs - with no colony growth. At the lower temperature, therefore, we used the strain to query the compatibility between the nematode Gα proteins ODR-3 and GPA-3 or chimaeras of these proteins with the last five amino acids of the yeast Gα; and the yeast Gβγ. Only Gα subunits or chimaeras that can bind to yeast Gβγ can sequester it and restore growth at the lower temperature, which is therefore a clear indication of functional interaction between endogenous yeast Gβγ and the test Gα construct. This assay is conducted in the absence of alpha-mating peptide and is valid whether or not a particular Gα is capable or incapable of binding the STE2 GPCR.

**Figure 1:**
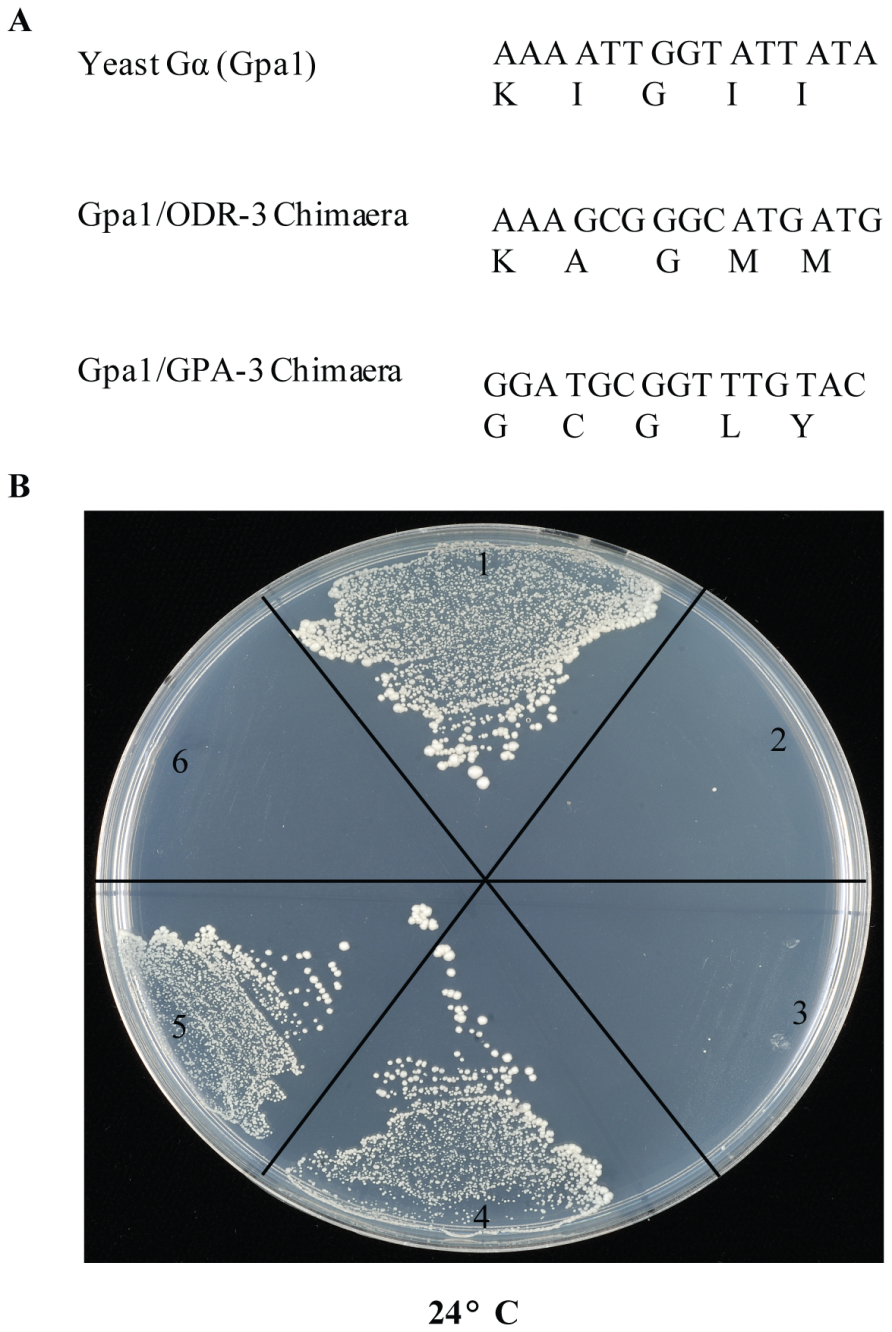
Characterisation of yeast/*C. elegans* Gα chimaeras. **A**. Sequences of the five C-terminal amino acids of Gα proteins used in this study. **B**. Complementation of growth arrest due to Gpa-1 t^s^ mutation with Gpa1/*C. elegans* Gα chimaeras at 24°C. All constructs grow at 34°C as shown in Figure S3. In the panel: **1**. Endogenous yeast Gpa1 used as a positive control. **2**. *C. elegans* GPA-3 fails to rescue the growth at 24°C. **3**. *C. elegans* ODR-3 fails to rescue the growth at 24°C. **4**. Gpa1/*GPA-3* chimaera complements the Gpa-1 t^s^ mutation. **5**. Gpa1/*ODR-3* chimaera complements the Gpa-1 t^s^ mutation. **6**. Vector-alone used as a negative control.

**Figure 2:**
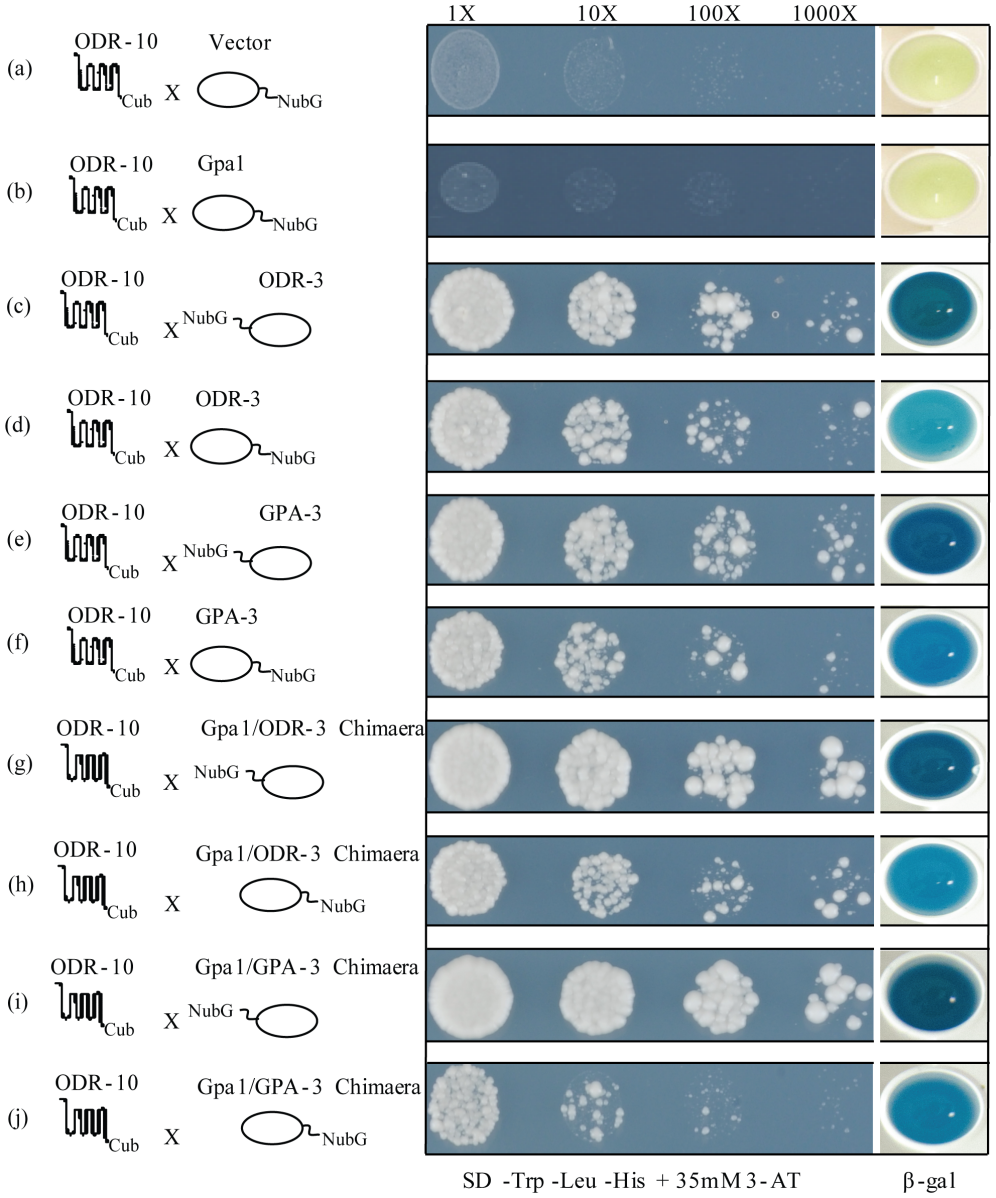
ODR-10 interaction with Gpa1/ODR-3 chimaera and Gpa1/GPA-3 chimaera in the split-ubiquitin yeast two-hybrid system. (**a**) ODR-10 does not interact non-specifically with NubG. (**b**) ODR-10 does not interact with yeast Gpa1. (**c**) and (**d**) ODR-3 couples to ODR-10 either NubG attached to N- or C-terminus. (**e**) and (**f**) GPA-3 couples to ODR-10 either NubG attached to N- or C-terminus. (**g**) and (**h**) Gpa1/ODR-3 chimaera couples to ODR-10 either NubG attached to N- or C-terminus. (**i**) and (**j**) Gpa1/GPA-3 chimaera couples to ODR-10 either NubG attached to N- or C-terminus. Yeast transformants contained both a Cub fusion and a NubG fusion construct were grown on drop out media (SD -Leu and -Trp) (Figure S4) and selective medium lacking histidine (SD -Leu, -Trp and -His) containing 35 mM 3-Amino-1,2,4-triazole (3-AT). β-galactosidase assays were performed to verify interactions. Cells were spotted as one-tenth dilutions starting at Abs_600 nm_ 1.

We placed each of the two main *C. elegans* olfactory Gα proteins, ODR-3 and GPA-3, under the control of the native yeast *GPA1* promoter, to avoid over or under expression of the Gα, and transformed YGS5 yeast cells with plasmids encoding them. Both transformants grew at 34°C (Figure S3) because *STE11* is inactivated by the *ts* allele. In contrast, at 24°C, cells containing *odr-3* and *gpa-3* did not grow (Figure 1), whereas the *GPA1* positive control cells were still able to grow. We concluded that, the nematode Gα subunits were unable to complement the yeast *GPA1* deletion, unlike some mammalian and rat Gα-proteins [34], [35]. The failure to complement, indicates either that ODR-3 and GPA-3 were not expressed or that they could not bind to the yeast Gβγ complex. However, Figure 2 demonstrates the observation that ODR-3 (2c & 2d) and GPA-3 (2e & 2f), modified with N-terminal or C-terminal NubG, express just as well as the equivalent versions of the chimaeras (2g-j), which suggests that it is a failure to bind, rather than a failure to express, that underlies the failure to rescue growth of YGS5.

Previous studies have shown that the amino-terminus of the Gα protein subunit is important for interacting with the Gβγ complex, whereas the last five amino acids of the carboxyl-terminus are important for interacting with the receptor [14]–[16], [39]. We generated yeast-*C. elegans* chimaeric Gα subunits, in which the carboxyl-terminal five amino acid residues of the endogenous yeast Gα subunit (Gpa1) were substituted with those from the *C. elegans* ODR-3 or GPA-3 Gα subunits, generating the chimaeras *GPA1/odr-3* and *GPA1/gpa-3*, which we placed downstream of the *GPA1* promoter (Figure 1A). Both Gpa1/ODR-3 and Gpa1/GPA-3 chimaeras rescued growth of the YGS5 yeast mutant strain at 24°C (Figure 1B), confirming that they can successfully couple with the yeast Gβγ complex.

In order to ensure consistency for long term use and simplify the number of simultaneous selections required, we used homologous recombination of *GPA1/odr-3* and *GPA1/gpa-3* chimaeras to replace the native *GPA1* yeast locus in our Cyb yeast strain. For this purpose, constructs *GPA1/odr-3-*PpURA3 and *GPA1/gpa-3*-PpURA3 were linearised with *Spe*I and transformed into the Cyb yeast strain. The transformants were selected on drop out media without uracil, yielding the Cyb *GPA1/odr-3* and Cyb *GPA1/gpa-3* chimaeric strains, and the integrations were confirmed by PCR.

### ODR-10 interaction with GPA-1/ODR-3 and GPA-1/GPA-3 chimaeras

Having modified nematode Gα proteins to allow them to interact with yeast Gβγ it was also important to check whether the modified proteins can still interact with the nematode GPCRs. We used the split-ubiquitin membrane yeast two-hybrid system (SUY2H) [40]–[45] to test for interaction between ODR-10 and the chimaeric Gα subunits. In the SUY2H system, proteins of interest are fused to either the N- or C-terminal moiety of a mutated ubiquitin. The N-terminus of split-ubiquitin includes an Ile3 to Gly3 mutation (NubG), which substantially reduces NubG's affinity for Cub when expressed in the same cells. The C-terminal moiety of the split-ubiquitin includes an artificial transcription factor domain (Cub-LexA-VP16). Upon *in vivo* interaction of their respective protein fusion partners, NubG and Cub are forced into close proximity and the weak interaction can be detected by the release of the LexA-VP16 transcription factor, inducing transcriptional activation of growth or colorimetric reporter genes e.g. *HIS3*, *ADE2*, and *lacZ*. The split-ubiquitin system is therefore useful for investigating protein interactions between GPCRs and Gα subunits [43], [45].

It is believed that, *in vivo*, ODR-10 can physically interact with both ODR-3 and GPA-3 as they act redundantly in *C. elegans* olfactory signalling [9], [20], [21]. *Odr-3*, *gpa-3* and *odr-10* genes were therefore used to test the SUY2H system. In the presence of 35 mM 3-amino-1, 2,4-triazole (3-AT), which is required to establish an appropriate level of stringency, ODR-10-Cub showed neither growth nor *lacZ* expression when co-expressed with yeast Gpa-1 NubG, confirming our previous observation that ODR-10 does not bind appreciably to the native yeast Gα subunit, Gpa1 (Figure 2b). On the other hand, ODR-10-Cub showed growth and strong *lacZ* expression when co-expressed with NubG-ODR-3, NubG-GPA-3, a NubG-Gpa1/ODR-3 chimaera or a NubG-Gpa1/GPA-3 chimaera (Figure 2c, 2e, 2g and 2i), suggesting strong interactions between ODR-10 and the nematode Gα subunits and the chimaeras derived from them. Growth was also observed with the inverted ubiquitin fusions, i.e. when ODR-10-Cub protein was co-expressed with ODR-3-NubG, GPA-3-NubG, Gpa1/ODR-3-NubG or Gpa1/GPA-3-NubG (Figure 2d, 2f, 2h and 2j), albeit it at lower levels. In these cases, the level of *lacZ* expression was also reduced. This may indicate that fusion of NubG to the C-terminus of the Gα hinders its interaction with the GPCR. A negative control expressing ODR-10-Cub with NubG alone (pPR3-STE) showed no growth (Figure 2a). Co-expression of ODR-10-Cub with wild-type Nub I (Ost1-NubI) served as a positive control and resulted in growth on His-deficient medium and expression of the *lacZ* reporter (Figure S4Ba) confirming that ODR-10 is functionally expressed in this system.

### Localisation of GFP^2^ tagged *C. elegans* ODR-10 receptor in the Cyb yeast strain

**Figure 3:**
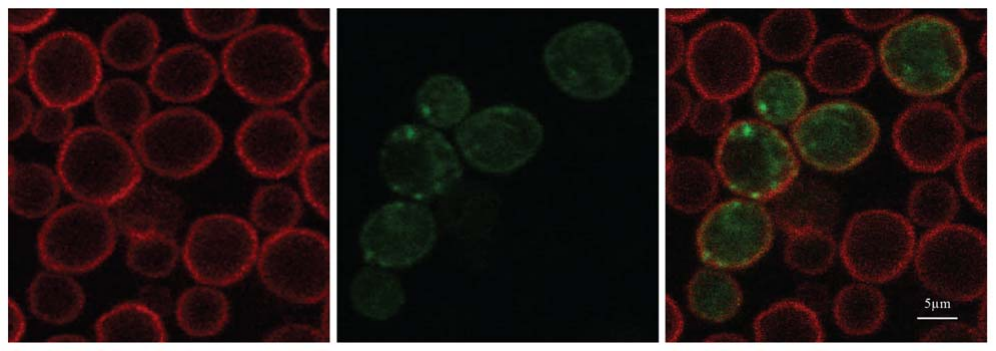
Expression of GFP^2^ tagged olfactory receptor ODR-10 in the Cyb yeast mutant. From left to right, Cyb cells stained with Evans Blue– a plasma membrane specific dye; GFP^2^ signal and plasma membrane localisation; overlay of two previous images. Scale bar = 5 µm.

A GFP^2^ tag was introduced into the third intracellular loop of ODR-10 and expressed in the Cyb yeast strain. A single colony was used to express GFP^2^ tagged ODR-10 under the specific microscopic conditions used (see Materials and Methods) with forty-three percent of yeast cells observed to express GFP^2^. Although some of ODR-10 was localised to the yeast plasma membrane, much of it was localised intracellularly (Figure 3), similar to what has previously been observed for mammalian chemoreceptors [18]. It has also been shown that mammalian ORs and *C. elegans* ODR-10 can function whether they are localised to yeast ER, Golgi or plasma membrane [24], [46]. On this basis, we believe it is likely that ODR-10 can functionally couple intracellular signalling to ligand stimulation, as we subsequently show, notwithstanding the receptors predominant expression in intracellular membranes. A previous study from our lab confirmed by western blotting that full-length ODR-10 protein is the only expression product in yeast that immunostains with a specific anti-ODR-10 antibody[24]. Furthermore, in the same study we failed to observe any degradation or cleavage of GFP from ODR-10 conjugated to both a luciferase and GFP.

### Selection of an appropriate reporter

In yeast, a variety of reporter systems are available, including absorbance (*lacZ*), fluorescence (GFP), luminescence (*luciferase*) and growth (histidine), to monitor GPCR activation [47]. GFP and *luciferase* have both been used to detect signalling with mammalian GPCRs [16]–[18], [29]. In our hands, GFP was inconsistent and unreliable for detecting nematode OR activation (data not shown). β-galactosidase (*lacZ)* has also been successfully used for reporting GPCR activation and is suitable for a quantitative assay [14]. The *lacZ* reporter marker was cloned under a pheromone responsive promoter for quantitative assessment of reporter activity as described below.

### Selection of an appropriate promoter for *lacZ* reporter

**Figure 4:**
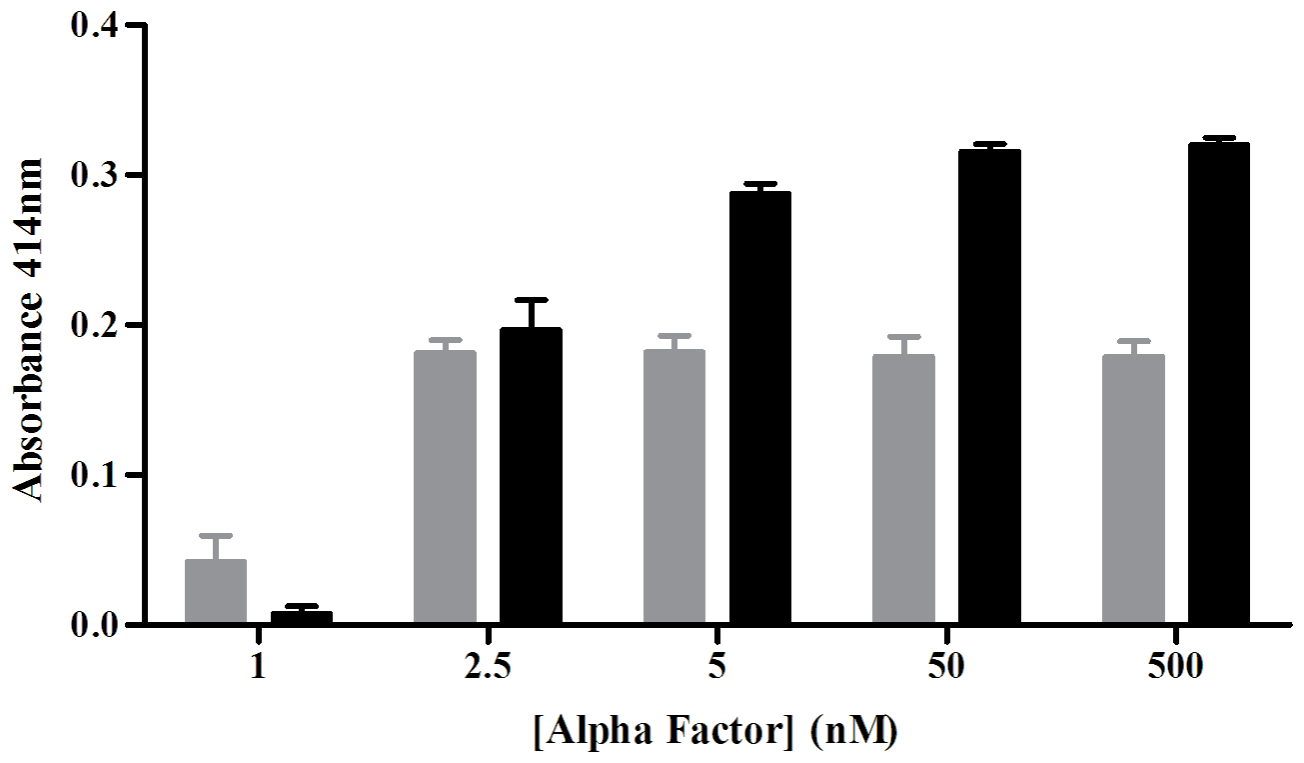
Comparison of efficiency and signal to noise ratio of *FUS1* and *FIG2* promoters in driving β-galactosidase reporter expression. α-factor sensing using the *lacZ* reporter gene assay in yeast cells (*ste2Δfar1Δsst2Δ* triple deletion alleles in the Cyb yeast strain) expressing the *PGK1: STE2*. β-galactosidase activity was measured in the yeast strains by stimulating with different concentrations of α-factor. *PGK1*: *STE2* and *FUS1: lacZ* (grey bars); *PGK1: STE2* and *FIG2: lacZ* (black bars). The mean pseudo Z-factor value calculated for *FUS1* promoter was 0.366 and for *FIG2* promoter 0.88 based on three biological repeats. Values are mean and standard deviations of three biological repeats.

We expressed the endogenous yeast Ste2 receptor in the Cyb strain under the constitutive *PGK1* promoter and compared ligand-induced *lacZ* expression using two different pheromone-responsive promoters, *FUS1* and *FIG2* [48]–[50]. The *FUS1* promoter has been used frequently to detect signal transduction by heterologously expressed GPCRs [14], [18], [29], [51] whereas the *FIG2* promoter had not been used in this system before. Both strains (*FUS1:lacZ*, *PGK1:STE2* and *FIG2:lacZ*, *PGK1:STE2*) showed dose-dependent expression of the *lacZ* reporter after activation with the Ste2 ligand, α-mating peptide (Figure 4). Pseudo Z-factor values [52]–[54] calculated for the *FUS1* and *FIG2* promoters were 0.366 and 0.88, respectively, based on three independent yeast transformants, each processed and measured in triplicate (Figure 4). The Z-factor coefficient is a simple statistical parameter that is commonly used to assess the quality of high throughput screening (HTS) assays [53]–[55]. A Z-factor score >0.5 is an indication of an excellent assay [56], [57]. In this case, we use the term pseudo Z-factor because our assay did not encompass the full range of variation seen when multiple ligands are screened against a receptor. Nevertheless, the comparison of the pseudo Z-factors, determined under identical conditions, shows that the *FIG2* promoter is substantially better than *FUS1* in this assay.

### Transducing odorant activation of nematode ORs in yeast using the β-galactosidase assay

Results to this point suggested that the nematode/yeast chimeric Gα subunits are a viable means of coupling nematode chemoreceptors to the yeast MAP kinase signalling pathway and that a *lacZ* reporter under the *FIG2* promoter would be a suitable reporter. We next sought to demonstrate specific odorant activation of the pathway.

**Figure 5:**
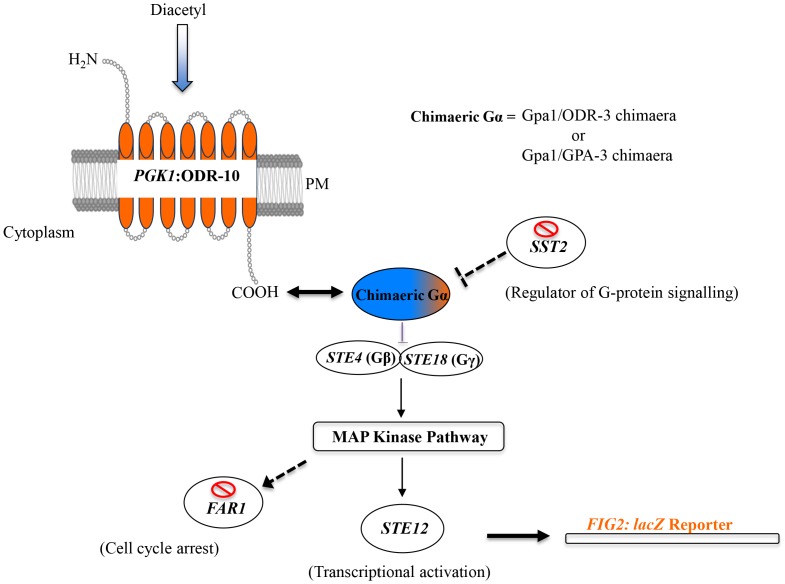
Schematic illustration of engineered yeast pheromone signalling pathway for analysing nematode ORs. In this pathway, the endogenous yeast GPCR, Ste2 was knocked out and replaced with a nematode OR (ODR-10), which induces signalling when activated by its ligand diacetyl. As nematode Gα subunits (ODR-3 and GPA-3) have low affinity for the yeast Gβγ, Gpa1/ODR-3 and Gpa1/GPA-3 chimaeras have been developed to incorporate receptor-binding properties of nematode subunits into the Gpa1 subunit that interacts with the yeast signalling machinery. Gene disruption was used to knock out *SST2* (to enhance signalling) and *FAR1* (to prevent cell cycle arrest). Signal activation was monitored by transforming *lacZ* reporter under *FIG2* promoter.

**Figure 6:**
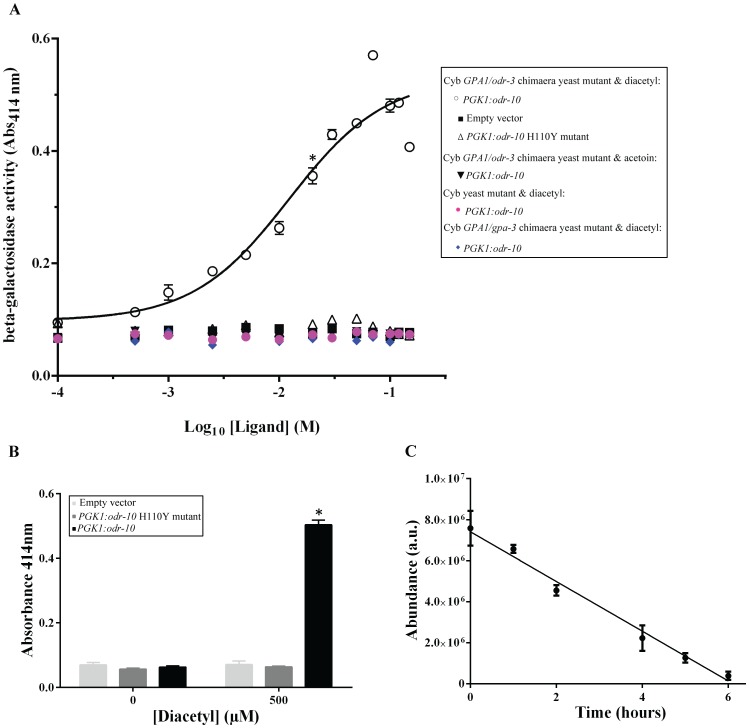
Concentration-response profile for odorant stimulated reporter gene expression. **A**. Expression of *lacZ* reporter gene driven by the *FIG2* promoter in Cyb yeast mutant containing different chimaeric Gα subunits. Values represent means ± SD of experiments performed on three independent transformants. The significance of the Cyb *GPA1/odr-3* chimaera *PGK1:odr-10* response was tested using a student t-test at the 100 µM concentration by direct comparison with the Cyb *GPA1/odr-3* chimaera *PGK1:odr-10* H110Y mutant. * p<0.01 **B**. Expression of *lacZ* reporter gene driven by the *FIG2* promoter in Cyb *GPA1/odr-3* chimaera yeast mutant in 96 well plate. Values represent means ± SD of experiments performed on three independent transformants. The significance of the Cyb *GPA1/odr-3* chimaera *PGK1:odr-10* response was tested using a student t-test at the 500 µM concentration by direct comparison with the Cyb *GPA1/odr-3* chimaera *PGK1:odr-10* H110Y mutant. * p<0.01 **C**. Change in abundance of diacetyl in the Cyb *GPA1/odr-3* chimaera yeast mutant containing receptor construct *PGK1:odr-10* and *FIG2: lacZ reporter* at different incubation times. Starting concentration of diacetyl in culture was 700 µM. Error bars are the standard error of three measurements.

The Cyb *GPA1/odr-3* chimaera and Cyb *GPA1/gpa-3* chimaera strains were co-transformed with an expression plasmid containing the model receptor *PGK1*:*odr-10* and the *FIG2: lacZ* reporter plasmid (Figure 5). Both strains expressing the ODR-10 receptor were exposed to the known ODR-10 ligand, diacetyl, and β-galactosidase activity was measured using ortho-nitrophenyl-β-galactoside (ONPG) as a substrate. Significant diacetyl-dependent increases in β-galactosidase activity were observed for the Cyb *GPA1/odr-3* chimaera strain compared with negative controls expressing a non-functional ODR-10 mutant (*PGK1*:*odr-10* H110Y) or vector alone (Figure 6A). A negative control ligand, 3-hydroxybutanone (acetoin) [10] did not stimulate increased β-galactosidase activity (Figure 6A). In contrast, with the Cyb *GPA1/gpa-3* chimaera yeast strain containing ODR-10, no β-galactosidase activity was observed after exposure to diacetyl (Figure 6A) suggesting that, although *Gpa1/GPA-3* chimaera interacts with ODR-10 (Figure 1), this interaction is not strong enough to activate the pathway in yeast.

The calculated EC_50_ value for diacetyl using the current assay was 0.11 mM (Figure 6A), which is less sensitive compared with the ODR-10 receptor in its native or directly transduced environments. Because ligand induction continues for many hours, we investigated whether and how quickly diacetyl concentration declines during the incubation. Starting from 700 µM, the diacetyl concentration decreased linearly with time (Figure 6C) and by seven hours no detectable diacetyl remained. The time averaged concentration of diacetyl, over seven hours, was 350 µM. Notwithstanding the degradation of diacetyl, the EC_50_ for diacetyl with ODR-10, is much higher in our yeast assay than *in vivo*, or indeed using other heterologous expression/transduction systems (as summarised in [24]). For example, although sensitivity cannot be measured directly in the whole nematode chemotaxis assay [58] it has been estimated to be in the parts per billion range [24]. The differences among methods may be due to differences in the membrane environments [59] but whole-cell assay systems are generically less sensitive, possibly because of limitations imposed by the cells' intrinsic transduction cascade [24].

### Optimisation of assay for high-throughput screening

The principle motivation for developing the assay was to enable de-orphaning of *C. elegans* chemoreceptor GPCRs. To enable rapid screening of a single GPCR with different ligands or the same ligand at different concentrations or a single ligand versus many GPCRs, we optimised the assay for a 96 well plate (See Materials and Methods for detail). Yeast cells were exposed to 500 µM diacetyl with shaking at 900 rpm for 17–20 hrs. Diacetyl specific induction of *lacZ* was observed (Figure 6B), whereas a strain expressing H110Y, the mutant ODR-10 receptor and vector alone did not show increased *lacZ* expression. Notwithstanding the relatively low sensitivity of the assay, its advantages of simplicity, cost and time effectiveness make it highly suitable for broad-scale screening.

### Conclusion

In this study, we have constructed and tested a robust assay for analysis of *C. elegans* GPCR function using yeast and the *lacZ* reporter marker. We confirmed previous genetic evidence for interaction between ODR-10 and the *C. elegans* Gα proteins, ODR-3 and GPA-3 using a split-ubiquitin yeast two-hybrid system and we were also able to demonstrate that the interaction was maintained in Gpa1 chimaeras incorporating only the C-terminal 5 amino acids of the nematode proteins. We found that unlike mammalian Gα proteins, *C. elegans* Gα proteins, ODR-3 and GPA-3 are unable to bind to yeast Gβγ. However Gpa1/*C. elegans* Gα chimaeras successfully complemented the Gpa1 mutation indicating they are able to bind to the yeast Gβγ. Finally, by replacing the endogenous yeast Gα protein, Gpa1, with OR-specific Gα chimaeras, the optimised yeast strain expressing ODR-10 showed concentration-dependent report expression induced by diacetyl. The *FIG2* promoter was found to optimally couple the expression of the reporter marker to ligand activation with much less background expression than the *FUS1* promoter used with mammalian olfactory receptors. With this heterologously engineered yeast system, we aim to accelerate the de-orphaning of *C. elegans* chemoreceptor GPCR proteins.

## Supporting Information

Figure S1 Schematic illustration of *GPA1/odr-3* and *GPA1/gpa-3* chimaeras cassettes inserted into Cyb yeast mutant at *GPA1* locus. In the figures, *GPA1* flanking DNA 1 is *GPA1* terminator and *GPA1* flanking DNA 2 is sequence from *NEM1* gene located downstream of *GPA1* in the yeast genome.
(TIF)

Figure S2 Schematic illustration of pDEST PGK-HIS plasmid.
(TIF)

Figure S3 Positive controls for Figure 2. All constructs grow at 34°C.
(TIF)

Figure S4 A. Positive controls for Figure 1. Yeast transformants containing both a Cub fusion and a NubG fusion construct were grown on drop out media (SD -Leu and -Trp) to test the presence of both constructs in yeast cells. Cells were spotted as one-tenth dilutions starting at Abs_600 nm_ 1. B. Controls used in the study. (**a**) ODR-10 couples to wild-type NubI which ensures the correct topology of the fusion protein. (**b**) Type 1 integral membrane protein amyloid A4 precursor protein (APP) couples to amyloid beta A4 precursor protein-binding family B member 1 (Fe65) to ensure that the assay is working.
(TIF)

Table S1 Primers used in this study.
(DOCX)
