# Supplementary figures and images for: Functional Coupling of a Nematode Chemoreceptor to the Yeast Pheromone Response Pathway
Source: PLoS One. 2014 Nov 21;9(11):e111429. doi: 10.1371/journal.pone.0111429 (PMC4240545; doi:10.1371/journal.pone.0111429)

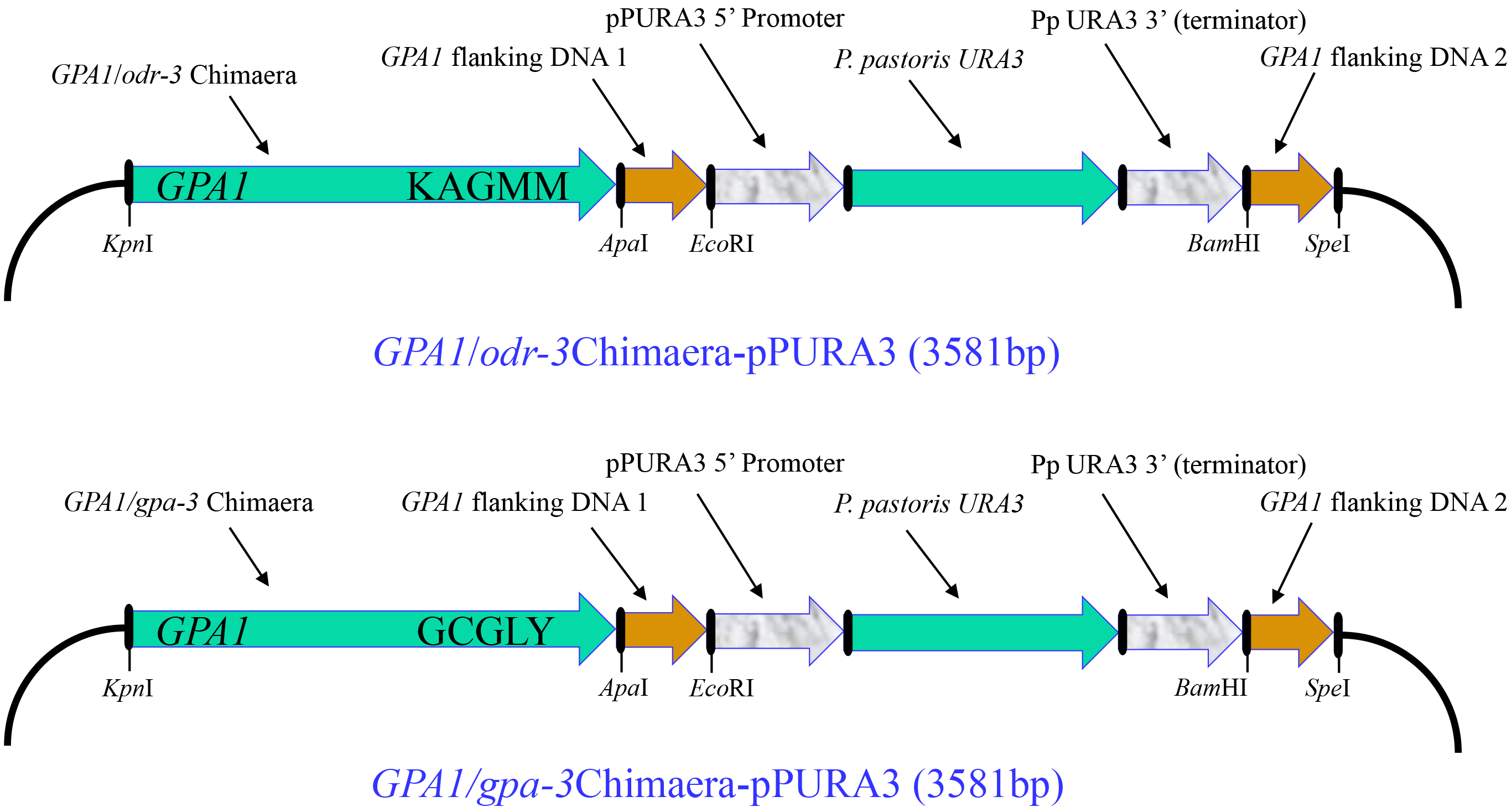

Supplement: Figure S1 — Schematic illustration of GPA1/odr-3 and GPA1/gpa-3 chimaeras cassettes inserted into Cyb yeast mutant at GPA1 locus. In the figures, GPA1 flanking DNA 1 is GPA1 terminator and GPA1 flanking DNA 2 is sequence from NEM1 gene located downstream of GPA1 in the yeast genome. (TIF) [file pone.0111429.s001.tif]

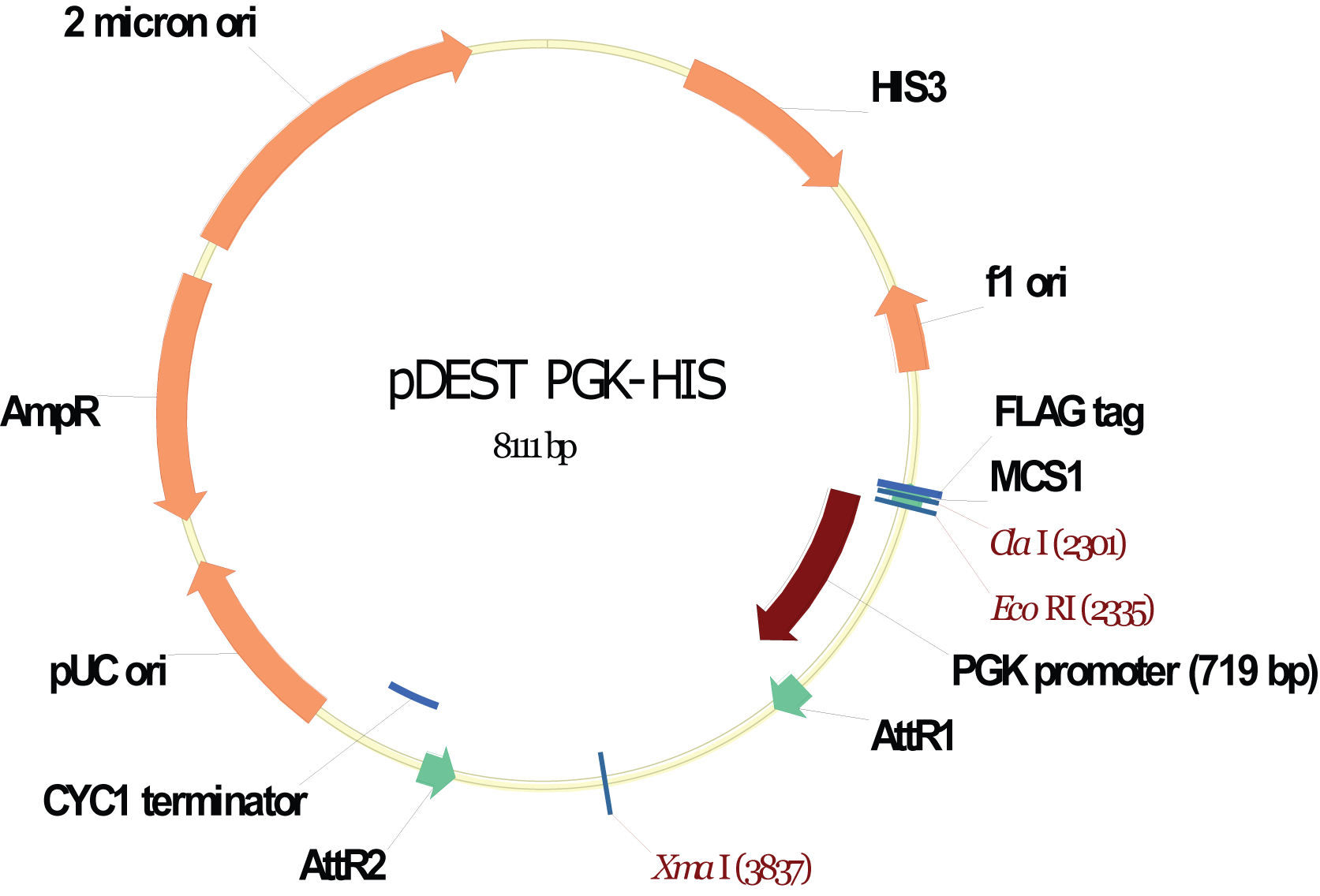

Supplement: Figure S2 — Schematic illustration of pDEST PGK-HIS plasmid. (TIF) [file pone.0111429.s002.tif]

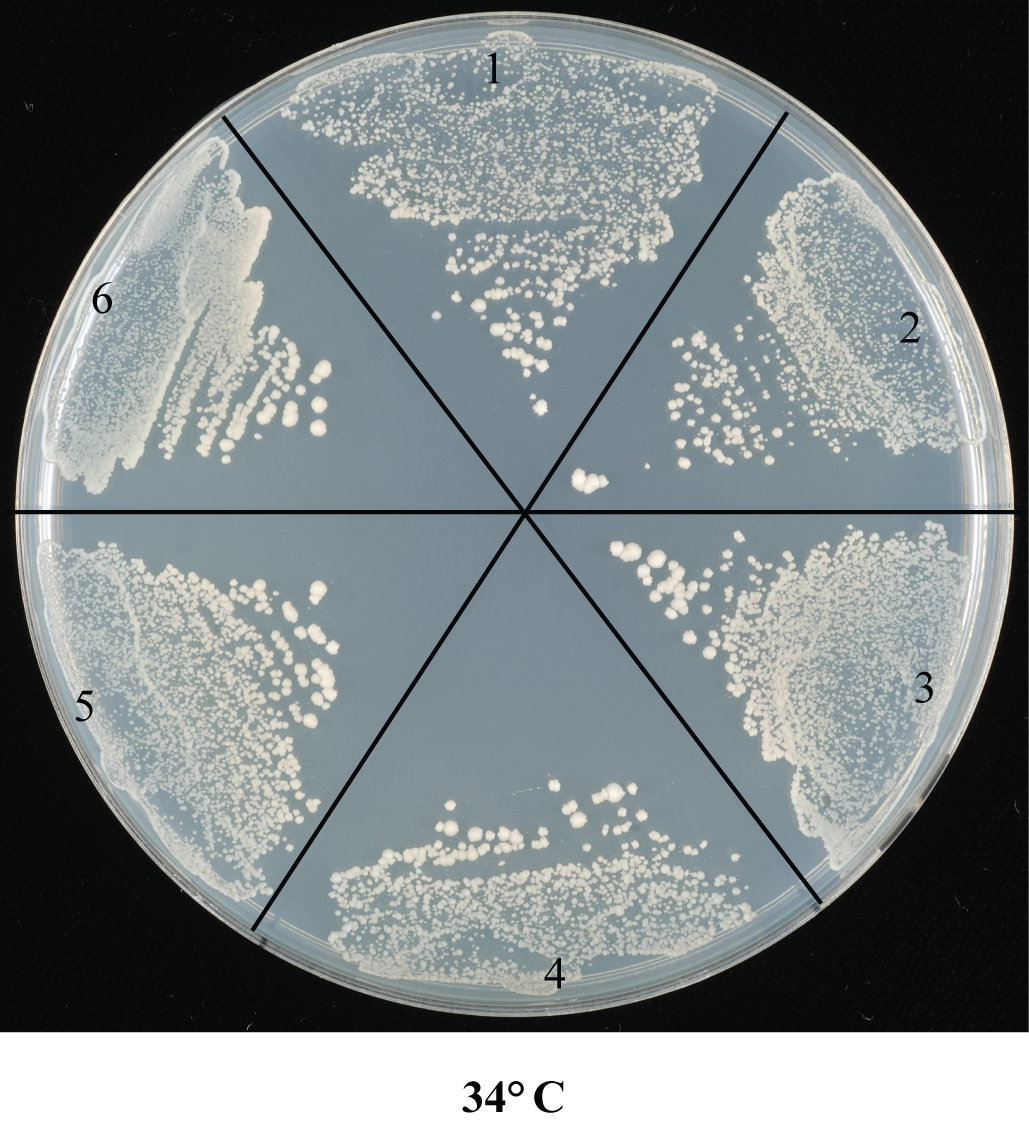

Supplement: Figure S3 — Positive controls for Figure 2. All constructs grow at 34°C. (TIF) [file pone.0111429.s003.tif]

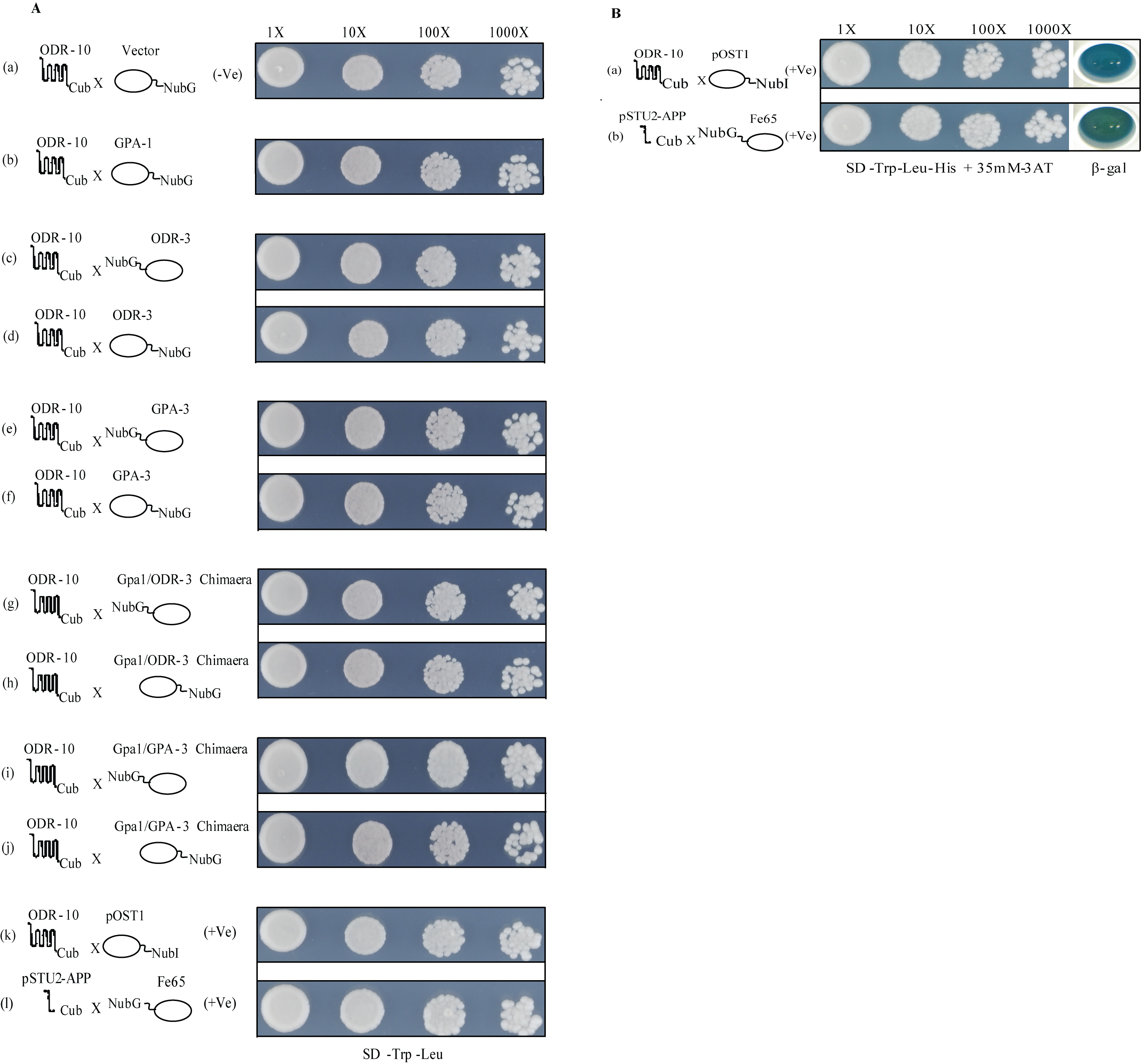

Supplement: Figure S4 — A. Positive controls for Figure 1. Yeast transformants containing both a Cub fusion and a NubG fusion construct were grown on drop out media (SD -Leu and -Trp) to test the presence of both constructs in yeast cells. Cells were spotted as one-tenth dilutions starting at Abs600 nm 1. B. Controls used in the study. (a) ODR-10 couples to wild-type NubI which ensures the correct topology of the fusion protein. (b) Type 1 integral membrane protein amyloid A4 precursor protein (APP) couples to amyloid beta A4 precursor protein-binding family B member 1 (Fe65) to ensure that the assay is working. (TIF) [file pone.0111429.s004.tif]
