# Supplementary material for: Functional Coupling of a Nematode Chemoreceptor to the Yeast Pheromone Response Pathway
Source: PLoS One. 2014 Nov 21;9(11):e111429. doi: 10.1371/journal.pone.0111429 (PMC4240545; doi:10.1371/journal.pone.0111429)
Supplement: Table S1 — Primers used in this study. (DOCX) [file pone.0111429.s005.docx]

| **Name** | **Oligonucleotide primer pair sequences** |
| --- | --- |
| SSt2F KO  SSt2R KO  SST2F Diag RIF2  SST2R Diag pAG25  FAR1F KO  FAR1R KO  FAR1F Diag SSY5  Far1F Diag SSY5  PGKprom-719FWD-EcoRI  PGKprom-1REV-ApaI  ccdB-For-ApaI  ccdB XhoI-Rev  Gpa1F Kpn1  Gor3ChiR Apa1  Gga3ChiR Apa1  Gpa1tF Apa1  Gpa1tR EcoRI  URA3F EcoRI  URA3R BamHI  Nem1F BamHI  Nem1R SpeI  GPA1pF AgeI  GPA1pR NotI  Odr3F NotI  Odr3R ClaI  Gpa3F NotI  Gpa3R ClaI  GPA1F NotI  Gpa1R ClaI  Gor3R ClaI  Gga3R ClaI  Fig2F BamHI  Fig2R NcoI  LacZF NcoI  LacZR SpeI | 5’-TTTTGCACGCACTATCTGAGGCGTTATAGGTTCAATTTGGTAATTAAAGATAGAGTTGTAAGCAGCTGAAGCTTC​GTACGCTGCAGG-3’  5’-CTAAAGAAAAAAAAAAGGACTGTTTGTGCAATTGTACCTGAAGATGAGTAAGACTCTCAATGGCCGCATAGGCCAC​TAGTGGATCTG-3’  5’-CAGTCTTACAACTCGCTTGTCACAT-3’  5’-AAGAGTGGTACCCATGGTTGTTTATG-3’  5’-CCTTTACACAAAGTCTATAGATCCACTGGAAAGCTTCGTGGGCGTAAGAAGGCAATCTATTACAGCTGAAGCTTCGT​ACGCTGCAGG-3’  5’-CACCCGCAGCCATATCCCCCAAATATACTTGCTCGAATGTAGCTTGTGGTAGACGAAGAGGCCGCATAGGCCACTAGTGGATCTG-3’  5’-GCTTCTACGGGATACACTTCA-3’  5’-GCTTCTACGGGATACACTTCA-3’  5’-GGCCGAATTCCATTTGCAAGAATTACTCGTGA-3’  5’-GGCCGGGCCCTGTTTTATATTTGTTGTAAAAAGTAG-3’  5’-CCCCGGGCCCACAAGTTTGTACAAAAAAGCTGA-3’  5’-CCGGCTCGAGACCACTTTGTACAAGAAAGCTGA-3’  5’-GATCGGTACCATGGGGTGTACAGTGAG-3’  5’-GATCGGGCCCTCACATCATGCCCGCTTTCTTAAGGTTTTGCTGGATG-3’  5’-GATCGGGCCCTTAGTACAAACCGCATCCTTTAAGGTTTTGCTGGATG-3’  5’-ATGCGGGCCCGTATAATTAAAGTAGTG-3’  5’-GATCGAATTCGAGATAATACCCTGTCCT-3’  5’-CATGGAATTCCTCTCTCACTGTGCTTGA-3’  5’-TAGCGGATCCGTTCGTTACTAGCCGTTCAA-3’  5’-CATGGGATCCTTCTCCCTGGTAAATACT-3’  5’-TAGCACTAGTCTAAGAAAATTCACCGAA-3’  5’-GATCACCGGTAAATGTGCATTAAAGCA-3’  5’-CATGGCGGCCGCTATTTCCTACCTTAATA-3’  5’-GATCGCGGCCGCATGGGCTCATGCCAGAGCAATG-3’  5’-CATGATCGATTTACATCATTCCTGCTTTTTGT-3’  5’-GATCGCGGCCGCATGGGATTATGCCAATC-3’  5’-GATCGAGCTCTTAGTACAAACCGCATCC-3’  5’-GATCGCGGCCGCATGGGGTGTACAGTGAG-3’  5’-CATGATCGATTCATATAATACCAATTTTTTTAAG-3’  5’-GATCATCGATTCACATCATGCCCGCTTTCTTAAGGTTTTGCTGGATG-3’  5’-GATCATCGATTTAGTACAAACCGCATCCTTTAAGGTTTTGCTGGATG-3’  5’-GATCGGATCCAATCACACGTCAGCGTCATG-3’  5’-GATCCCATGGTGCAGTTATATTCGGTAGATGATA-3’  5’-CATGCCATGGATGACCATGATTACGGATTCACTGG-3’  5’- GATCACTAGTTTATTTTTGACACCAGACCAACTGG-3’ |

**Table S1: Primers used in this study**
